# Coping strategies and depression during the COVID-19 pandemic in pregnant women: a cross sectional study

**DOI:** 10.1186/s12888-022-03792-8

**Published:** 2022-03-01

**Authors:** Mojgan Firouzbakht, Narges Rahmani, Hamid Sharif Nia, Shabnam Omidvar

**Affiliations:** 1 Department of Nursing and Midwifery, Comprehensive Health Research Center, Babol Branch, Islamic Azad University, Babol, Iran; 2 Department of Nursing and Midwifery, Comprehensive Health Research Center, Babol Branch, Islamic Azad University, Babol, Iran; 3 Department of Nursing, Amol Faculty of Nursing and Midwifery, Traditional and Complementary Medicine Research Center, Addiction Institute, Mazandaran University of Medical Sciences, Sari, Iran; 4 Department of Nursing and Midwifery, Social Determinants of Health Research Center, Health Research Institute, Babol University of Medical Sciences, Babol, Iran

**Keywords:** Coping strategies, Depression, Anxiety, Pregnancy, COVID-19

## Abstract

**Background:**

Pregnant women are vulnerable to psychological problems depending on the adaptive capacities of their personality and coping strategies. This study aimed to investigate the association between coping strategies of pregnant women during the COVID-19 pandemic and depression.

**Methods:**

This web-based cross-sectional study was conducted in 2021 on 318 pregnant women in Amol, Iran. Data collection was performed via questionnaires (Brief cope, Edinburgh Postnatal Depression Scale, CDA, and Demographic questionnaire). The questionnaires were completed through the WhatsApp and Telegram applications. Data were analyzed using the hierarchical regression analysis and SPSS software (v. 21) at the significance level of 0.05.

**Results:**

About 40% of participants had depression. The most prevalent coping strategy used by pregnant women was the avoidance strategy. Hierarchical regression revealed that the coping strategy of avoidance was a significant predictor of depression (β = 0.226, *p* = 0.046) after controlling background characteristics.

**Conclusions:**

The findings of this study suggest that avoidance style associated with depression in pregnant women. Therefore, obtaining further knowledge about impacts of coping strategies on pregnant women seems to be essential.

## Background

Although many women report that pregnancy is a joyful and happy period in their lives, pregnancy per se can be a stressful event. Pregnant women require numerous adjustments in their physiological, financial, occupational, and many other aspects of life in order to avoid emotional distress [1].

Psychological stress is experienced during pregnancy with negative consequences of maternal and fetus wellbeing [2]. For instance, women with high levels of stress may report increased levels of anxiety and develop symptoms of severe psychological syndromes, including depression and other psychological disorders [3]. Depression is the most common psychiatric morbidity in the pregnancy [4]. Antepartum depression is associated with adverse effects on mother and newborn [5]. However, cortisol will increase in the mothers with depression during pregnancy [6, 7]. Increased levels of glucocorticoids during the development of the fetus lead to a higher probability of adverse birth outcomes (e.g., preterm birth and intrauterine growth restriction), predisposition to be overweight, other late-onset diseases [8], and negative consequences of children’s cognitive, emotional, and behavioral development [2, 9].

Fortunately, not all women who have experienced stressful events show the adverse effects of anxiety. Using a resilience approach, one can shed light on this critically significant problem [10]. For example, various coping behavior and coping skills may lead to different psychological and physiological effects of stress exposure during pregnancy [11]. Coping is defined as frequently changing cognitive and behavioral efforts in dealing with the demands of particular stressful situations [12]. According to the transactional model of stress, appraisal, and coping, that offers a theoretical framework for assessing the processes of coping with stressful situations [12], when encountering a stressor, individuals, first, evaluate the potential threat (primary appraisal) and, then, assess their coping resources and options [13]. Regulation of stressful emotions (emotion-focused coping strategies such as passive and active avoidance, escaping, seeking social support, and positively reappraising the stressor) and management of the problem which causes the distress (problem-focused coping strategies such as planning how to change the stressor, seeking practical or informational support, and confronting the stressful situation) are two widely known major functions of coping [12].

With regard to pregnancy, coping efforts may have effects on birth outcomes through reducing or impeding negative emotional, cognitive, behavioral, and physiological responses to stressors. Consequently, an appropriate coping response can serve as a resilience resource for the pregnant mothers and their infants, thereby protecting them from the potentially detrimental impacts of prenatal stress exposure [8, 14]. For instance, coping in those who seek emotional support or take action to resolve the problem has fewer harmful effects of stress. In contrast, vulnerability will be heightened in those who choose avoidance strategies in dealing with the stressor or engage themselves in adverse health behaviors, such as smoking, to decrease distress [1]. Similar results have been found in pregnant women whose choice of avoidance coping has been consistently associated with poorer maternal and child health [15–17]. By contrast, the results regarding the effect of problem-focused and emotion-focused copings are mixed. Usually, these strategies can be associated with better maternal adjustment and child outcomes [1, 16, 18].

However, conceptual approaches found in the wide coping literature whose focus has been on coping as a moderator of the effects of stressors on health [12] suggest that the evaluation of coping with stress during pregnancy is beneficial. Some studies have investigated the impact of coping on the relationship between stress and maternal outcome during pregnancy. For instance, in one study black pregnant women who used an avoidance style to deal stress, were more likely experience psychological disorders [19]. Active coping in another study had no association with depression in women who were with or without gestational diabetes [20]. Also, optimism and resilience decrease levels of psychological distress among pregnant [21]. The anticipated stress-buffering impacts of coping resources (social support) and coping strategies (wishful thinking) on depression have not been supported by one other study [22]. However, as adaption of coping strategies is affected by sociocultural and spiritual beliefs and characteristics [23] research on coping strategies must be cultural- specific.

The COVID-19 pandemic which began in 2019 changed the circumstances of the world so rapidly, as a result of which many individuals, including pregnant women, experienced the loss of livelihood, increased financial burden, physical isolation, decreased personal support systems, professional services, and illness as well [24]. At present (Aug. 2021), more than 4,400,000 people have died globally from the pandemic, Iran is in fifth wave of COVID-19 and more than 100,000 of Iranian people have died. (https://covid19.who.int/). During the COVID-19 pandemic, pregnant women have been shown to experienced psychological distress. Several studies have reported higher levels of anxiety and depressive symptoms in pregnant women during the COVID-19 pandemic [25–28]. Pregnant women are vulnerable to psychological problems depending on the adaptive capacities of their personalities and coping strategies [29]. This situation provides opportunities to examine the effectiveness of different coping strategies pregnant women employ when encountering an uncontrollable event. To the best of our knowledge, no study has been conducted to investigate coping strategies during COVID-19 pandemic among pregnant women in Iran. The aim of the current study was to investigate coping strategies of pregnant women during COVID-19 pandemic and the relationship of these strategies with depression.

## Methods

### Study design, participants

This cross-sectional research was conducted from April 21 to May 30, 2021, in Amol a city in north of, Iran. Due to COVID-19 pandemic to prevent the spread of disease, the data was collected through an online survey.

This study population consisted of all pregnant women that had a file in primary healthcare centers in Amol, Iran. Pregnant women with healthy singleton pregnancy, minimum literacy, access to telephone and social media, no history of stressful events of life during the past 6 months (e.g., marriage, divorce, or significant loss), no mental health disorders, and no psychiatric medications intake were included. Voluntary withdrawal of the subjects from the study was the exclusion criterion. The samples of the study were selected purposively.

Women were recruited by midwives. After creating a list of eligible women and their telephone numbers, the midwives who were present in the centers called the women by the telephone and invited them to the study. This survey was released on website (https://survey.porsline.ir/#/survey/61851/build). Moreover, link of the questionnaire was provided to the women through the WhatsApp and Telegram applications, and they were asked to complete the questionnaires. Sample size for Linear multiple regression analysis (Fixed model, R2 deviation from zero) was estimated using the G*Power [30]. Accordingly, considering confidence level of 0.95, a power of 0.95, and an effect size of 0.1, for 12 predictors, and 10% missing sample size was determined to be 300.

### Measures

The study instruments included a demographic and obstetrics characteristics questionnaire, the Edinburgh Postnatal Depression Scale, Corona Disease Anxiety (CDA) Questionnaire, and the Brief Cope Questionnaire.

The demographic and obstetrics characteristics questionnaire consisted of age (years), educational level (Below diploma, diploma, college - university), place of residence (Urban, Rural), level of self -report family support (Low, Moderate, High), number of pregnancies, any history of problems during pregnancy (abortion, premature birth, bleeding during pregnancy, and pain).

### The Edinburgh postnatal depression

This scale is a self-report questionnaire that report objects’ feeling in the past week. This questionnaire is used to assess the level of depression both during pregnancy and after delivery (e.g., have been able to laugh and see the funny side of things). It contains ten items, each item scored in four-point scale from 0 to 3 based on the severity of symptoms. The items 1, 2, and 4 scored 0–3 and items 3 and 5–10 scored reversed (3–0). The scale’s total score is 0–30, and cutoff point the scale is 12.5. The persons who gain higher than 12.5 scores are supposed to be depressed [31]. The validity and reliability of the questionnaire was confirmed in Iranian population [32].

### The Corona disease anxiety questionnaire (CDA-Q)

This questionnaire includes 18 items, that measure anxiety corona disease in two physical and mental level (e.g., Thinking about Coronavirus makes me anxious). The items are based on a 4-point scale from 0 (*Never*) to 3 (*Always*) Total score range of the questionnaire is between 0 and 54, and cut off point of the questionnaire is 37.8. The respondents whose scores are higher than 37.8 are considered to be anxious. The CDA-Q has been developed in Iran. This questionnaire has acceptable validity and reliability [33].

### Brief-COPE

As a 28-item self-report questionnaire, the Brief-COPE has been designed to measure effective and ineffective methods of coping with a stressful life event (e.g., I’ve been turning to work or other activities to take my mind off things). Being used in healthcare settings, the scale determines the responses of patients to a serious diagnosis.

The women’s use of coping strategies was measured using the 28-item Brief COPE [34], that includes 14 specific coping strategies: subscales of denial (items 3, 8), substance use (items 4, 11), venting (items 9, 21), behavioral disengagement (items 6, 16), self-distraction (items 1, 19) and self-blame (items 13,26), positive reframing (items 12, 17), planning (items 14, 25), acceptance (items 20, 24), seeking emotional support (items 5, 15), and seeking informational support (items 10, 23), humor (items 18, 28) and religion (items 22, 27). The women were asked to rate the usual frequency of using coping strategies based on a 4-point rating scale ranging from 1 (*I did not do this at all*) to 4 (*I did this a lot*). The higher the scores, the stronger was the tendency to adopt coping behaviors with regard to events. The 14 subscales were classified into three categories [35]: problem-focused (Active Coping, Planning and Using Instrumental Support), emotion-focused (Positive Reframing, Acceptance, Humor, Religion and Using Emotional Support), and avoidance Coping (Self-Distraction, Denial, Venting, Substance Use, Behavioral Disengagement, and Self-Blame). The internal consistency reliability of the subscales was acceptable (αs = .50 to .90). The validity and reliability of this questionnaire were also acceptable [36].

### Data analysis

In order to summarize the socio-demographic and obstetrics characteristics of the participants, SPSS version 20 was used. Frequencies and percentages together with mean and standard deviations (SD) were used to summarize categorical and continuous variables respectively. Hierarchical multiple regression analysis was employed for examining the variability of depression following anxiety and three coping styles after controlling the demographic and obstetrics characteristics. Variance inflation factors (< 10) were evaluated in the regression models to ensure collinearity was not a problem in any proposed models. In order to examine the independent associations between anxiety, coping style, and depression, three separate multiple regression models were investigated. The control variables were entered in block one, anxiety in block two, and coping style in block three.

## Results

The questionnaire was viewed 768 times during 2 months, and 318 individuals completed the questionnaire. The response rate was 60%. The mean time to answer the questions was 15 min, and 97% of answers were via the WhatsApp application and 3% of answers were via Telegram.

The demographic characteristics are shown in Table 1.

**Table 1:** Participants’ demographic and obstetrics characteristics

Variable	Mean (SD)/ frequency (%)
Age	
< 20	19 (6%)
20–35	289 (85.2%)
> 35	28 (8.8%)
Education	
Below diploma	32 (10.1%)
Diploma	112 (35.2%)
University	174 (54.7%)
Place of residence.	
Urban	213 (67%)
Rural	105 (33%)
Social support	
Low	15 (4.7%)
Moderate	142 (44.7%)
High	161 (50.6%)
Number of pregnancies	
1	190 (59.7%)
> =2	128 (40.3%)
Problems during current pregnancy (abortion, Preterm labor, vaginal bleeding)	
No	243 (76.4%)
Yes	75 (23.6%)

The mean scores of the Edinburgh questionnaire and Corona Disease Anxiety questionnaire were 12.16 ± 3.43 and 12.24 ± 7.38, respectively. About 40% of the participants had depression, and about 19% had COVID anxiety. There was a significant relationship between depression and COVID anxiety (Table 2). The most frequent coping strategy used by pregnant women was the avoidance coping strategy (Table 3).

**Table 2:** Relationship between depression and corona anxiety disease in pregnant women

Variables	Corona anxiety disease		χ2	*df*	*P*	*OR* *(95% CI)*
	No	Yes				
Depression						
No	160 (64.3%)	24 (34.8%)	19,251	1	< 0.001*	3.371 (1.146–5.896)
Yes	89 (35.7%)	45 (65.2%)

**Table 3:** Descriptive statistics and correlations among primary study variables

Variable	Mean ± SD (Range)	Correlation coefficient			
		CDA	Emotion-focus strategy	problem-focus strategy	Avoidance-strategy
Depression	12.16 ± 3.43 (0–30)	.383*	.048	.046	.027
CDA	12.24 ± 7.38 (0–54)	–	.100	.099	.047
Emotion-focus strategy	19.55 ± 5.27 (10–40)	–	–	.741*	.814*
Problem-focus strategy	10.85 ± 3.47 (6–24)	–	–	–	.840*
Avoidance strategy	24.38 ± 6.87 (12–48)	–	–	–	–

*P < 0.001

Before hierarchical regression analyses, the independent variables were examined for collinearity. The results of the variance inflation factor suggested that the estimated bs were well established in the following regression models.

The results of the hierarchical regression predicting depression from demographic characteristics, anxiety, and coping strategies are shown in Table 3. The results of step one showed that the variance explaining (*R* ^2^), with demographics predictors, was equal to 0.06 (adjusted R^2^ = .036). In model 1, age, education, and place of residence predicted depression. Next, CDA scores were entered into the regression equation. The change in variance accounted for (D*R* ^2^) was equal to 0.124, demonstrating a statistically significant increase in variance compared to the step one model (DF_(2304)_ = 42.36, *p* < 0.001). In step three, coping strategies (avoidance strategy, emotional-focus strategy, problem-focus strategy) were entered into the regression equation. The change in variance explaining (D*R* ^2^) was equal to 0.091, showing a statistically significant increase in variance accounted above the variability contributed by the previous predicting variables entered in step two (DF_(1304)_ = 2.47, *p* = 0.046). In final gravida, CDA, and avoidance strategy predicted depression in pregnant women (Table 4).

**Table 4:** Hierarchical regression analysis of evaluating depression predictors

Measures	Model 1		Model 2		Model3		VIF
*Block 1*	B	Sig.	B	Sig.	B	Sig.	
Age							
< 20	− 3.214	0.011*	−2.476	.037*	−1.558	.051	1.10
20–35	− 1.230	0.084	− 1.312	.048*	− 1.275	.056	1.13
> 35	Ref.						
Education							
Below diploma	0.774	.247	.351	.597	.156	.816	1.30
Diploma	1.029	0.023*	.816	.054	.812	.056	1.30
University	Ref.						
place of residence	−.862	.045*	−.531	.190	−.500	.216	1.15
Job	.329	.561	.322	.542	.326	.538	1.11
Social support	.596	.073	.203	.520	.203	.527	1.12
Gravid (number of pregnancy)	.073	.817	.627	.056	.702	.031*	1.24
Problems during pregnancy	.121	.618	.076	.827	1.12	.523	1.23
***Block 2***							
CDA			.15	< 0.001**	0.146	< 0.001**	1.11
***Block3***							
Avoidance strategy					.226	.046*	4.95
Problem-focus strategy					.107	.131	3.73
Emotion-focus strategy					.082	.206	3.23
Adjusted R^2^	.036		.150		. 163		
***∆*** **R2**	.060		.124		.091		
F change	2.45		42.36		2.47		
P	.014		< 0.001**		0.046*		

*P < 0.05, **P < 0.001

## Discussion

This study aimed at examining the relationship between coping strategies during the COVID − 19 pandemic and depression in pregnant women. In this study prevalence of depression was 40%. In a systematic review study the prevalence of depression was reported from 5.2 to 40% [37]. Some situations such as social distancing and quarantining were related with depression in pregnant women during the COVID-19 Pandemic [28]. Results of some studies reported an increase in prevalence of depression during the COVID-19 pandemic in general population [38] and pregnant women [39]. The results of the study showed that the majority of pregnant women during the COVID-19 pandemic used avoidance coping strategies in the face of stress. Moreover, the use of these strategies has a significant association with depression among the sample. Although avoidance is not a long-term successful coping strategy, it may reduce stress levels in the short term by escaping the situation which has caused stress [40]. Li’s (2020) study showed that using both problem-focused and emotional - focused coping was better for Psychological status [41]. Guo et al. (2020) showed that using problem-focused coping decreased mental health problems and emotional -focused coping style increased mental disorders [42] In a study in Saudi-Arabia during COVID-19 emotional - focused coping related to increased depression, anxiety and sleep disorders in people [43]. Although people use the coping strategies in the face of stressful situation, but some of these strategies are related to increased psychiatric disorders.

Based on the results of several meta - analysis studies, pregnant women experience mental health problems during the COVID-19 pandemic [44, 45]. In line with prior researches in pre- COVID [1, 46], and during the COVID- 19 pandemic showing that specific coping strategies are associated with better/worse mental health outcomes in pregnant women [47]. In order to realize the relationship between psychological stress and the human capacity in dealing with challenges, coping strategies have to be widely considered [12]. Based on the findings of various studies in this area, the most consistent findings are avoidance coping styles or behaviors. The results of studies have been associated with many adverse mental health outcomes in pregnancy, including lower general psychological wellbeing, increased distress, higher depressed mood, more anxiety, higher perceived stress, less positive attitudes towards screening, and greater potentiality for child abuse [48–51]. These findings are in line with the findings of the present study in which the use of avoidance strategies led to depression.

The context of an individual’s life and the type of stressors are important predictors of the most effective coping strategy used to decrease levels of distress. Additionally, certain personality characters such as age, socio-economic status, and internal or external locus of control may have an effect on coping styles [40]. Furthermore, an individual’s adaptive resources, such as appropriate coping skills, social support, and optimism, have been documented as protective of that, through reducing stress to improve health outcomes [52, 53]. In this study, pregnant women experienced stress and anxiety during the COVID-19 pandemic due to conditions such as quarantine, unavailability of health systems, distance from friends and family, and feelings of loneliness, and the use of avoidance coping strategies in dealing with stress had jeopardized their mental health. Although avoidance is not a long-term successful coping strategy, it may reduce stress levels in the short term by escaping the situation which has caused stress [40]. In the study of Latendresse and Ruiz (2010), avoidance coping style was associated with higher maternal levels of the corticotrophin-releasing hormone of placental origin implicated in the timing of delivery and the etiology of preterm birth [54].

The results of the Wheeler (2020) study showed that beyond social support, pregnant women used numerous active coping strategies to cope with their feelings during the COVID-19 pandemic. This study showed that women who widely used avoidance coping strategy prior to the pandemic reported more perceived stress both before and after this period [55]. These findings are in line with the results of the present study that the majority of pregnant mothers used avoidance strategies to cope with stress during the COVID-19 pandemic, which predicted the likelihood of depression in them.

### Limitations and strengths

This study needs to be interpreted with some limitations. The data were self-report without external observation. Additionally, although the differences in background variables were controlled, some variables, especially personality traits, which might moderate the impact of coping, could not be controlled. Thus, the obtained results might be influenced by uncontrollable confounding. Therefore, future studies should be investigated after controlling these additional psychosocial factors. On the other hand, this study was done in a cross-sectional mode that does not show any causal relationship between variables, indeed, it is not possible to know whether coping strategies lead to depression or vice versa. A cohort study could help to elucidate whether coping strategies predispose women to depression or are a consequence of depression. Finally, we were unable to control for participants completing the study twice by re-clicking the link. The most important strength of this study was that it used web-based data collection without exposing participants to the COVID-19. Furthermore, the study included a sufficient sample size to provide sufficient statistical power.

### Conclusions

The present study showed that prevalence of depression in pregnant women was high, and pregnant women primarily used avoidance coping strategies in the face of stress. Although this coping style may decrease stress in the short time, it is necessary to obtain more knowledge about the long-term effects of coping strategies on pregnant women. It seems that a cohort study must be conducted to evaluate causality between coping strategies and depression. Overall, the results of this study can be used in mental health planning in pregnant women. All pregnant women should be screened for depression at least once during pregnancy. Pregnant women need training for stress coping. The health care provider should consider coping strategies in pregnant women to decrease feto-maternal poor outcomes.

## Data Availability

The datasets analyzed during the current study are available from the corresponding author on reasonable request.

## Abbreviations

COVID-19: Coronavirus disease 2019
CDA: Corona-disease anxiety
SPSS: Statistical Package for Social Sciences
SD: Standard Deviation

## References

[1] Guardino CM, Dunkel Schetter C. Coping during pregnancy: a systematic review and recommendations. Health Psychol Rev. 2014;8(1):70–94. 10.1080/17437199.2012.752659 PMC3904449 24489596

[2] Van den Bergh BR, van den Heuvel MI, Lahti M, Braeken M, de Rooij SR, Entringer S, Hoyer D, Roseboom T, Räikkönen K, King S, Schwab M (2020). Prenatal developmental origins of behavior and mental health: The influence of maternal stress in pregnancy. Neurosci Biobehav Rev..

[3] Gourounti K, Anagnostopoulos F, Lykeridou K (2013). Coping strategies as psychological risk factor for antenatal anxiety, worries, and depression among Greek women. Arch Womens Mental Health.

[4] Okagbue HI, Adamu PI, Bishop SA, Oguntunde PE, Opanuga AA, Akhmetshin EM (2019). Systematic review of prevalence of antepartum depression during the trimesters of pregnancy. Open Access Maced J Med Sci.

[5] Orta OR, Gelaye B, Bain PA, Williams MA (2018). The association between maternal cortisol and depression during pregnancy, a systematic review. Arch Womens Mental Health.

[6] Cardwell MS (2013). Stress: pregnancy considerations. Obstet Gynecol Surv.

[7] Coussons-Read ME, Okun ML, Nettles CD (2007). Psychosocial stress increases inflammatory markers and alters cytokine production across pregnancy. Brain Behav Immun.

[8] Barbosa-Leiker C, Smith CL, Crespi EJ, Brooks O, Burduli E, Ranjo S (2021). Stressors, coping, and resources needed during the COVID-19 pandemic in a sample of perinatal women. BMC Pregnancy Childbirth.

[9] Davis EP, Head K, Buss C, Sandman CA (2017). Prenatal maternal cortisol concentrations predict neurodevelopment in middle childhood. Psychoneuroendocrinology.

[10] Davis EP, Narayan AJ (2021). Pregnancy as a period of risk, adaptation, and resilience for mothers and infants. Dev Psychopathol.

[11] Dunkel Schetter C, Glynn LM (2011). Stress in pregnancy: empirical evidence and theoretical issues to guide interdisciplinary research.

[12] Lazarus RS, Folkman S. Stress, appraisal, and coping: Springer publishing company; 1984.

[13] Lazarus RS (2006). Stress and emotion: a new synthesis.

[14] Cohen S, Wills TA (1985). Stress, social support, and the buffering hypothesis. Psychol Bull.

[15] Zhu P, Hao J-H, Tao R-X, Huang K, Jiang X-M, Zhu Y-D (2015). Sex-specific and time-dependent effects of prenatal stress on the early behavioral symptoms of ADHD: a longitudinal study in China. Eur Child Adolesc Psychiatry.

[16] Chen T, Laplante D, Elgbeili G, Brunet A, Simcock G, Kildea S (2020). Coping during pregnancy following exposure to a natural disaster: the QF2011 Queensland flood study. J Affect Disord.

[17] Oni O, Harville EW, Xiong X, Buekens P (2012). Impact of coping styles on post-traumatic stress disorder and depressive symptoms among pregnant women exposed to hurricane Katrina. Am J Disaster Med.

[18] Lau Y, Wang Y, Kwong DHK, Wang Y (2015). Testing direct and moderating effects of coping styles on the relationship between perceived stress and antenatal anxiety symptoms. J Psychosom Obstet Gynecol.

[19] Giurgescu C, Misra DP (2018). Psychosocial factors and preterm birth among black mothers and fathers. MCN: the American journal of maternal/child. Nursing.

[20] Spirito A, Ruggiero L, Bowen A, McGarvey S, Bond A, Coustan D (1991). Stress, coping, and social support as mediators of the emotional status of women with gestational diabetes. Psychol Health.

[21] McNamara J, Townsend ML, Herbert JS. A systemic review of maternal wellbeing and its relationship with maternal fetal attachment and early postpartum bonding. PLoS One. 2019;14(7). 10.1371/journal.pone.0220032 PMC6657859 31344070

[22] Pakenham KI, Smith A, Rattan SL (2007). Application of a stress and coping model to antenatal depressive symptomatology. Psychol Health Med.

[23] Roger G, Tweed LGC, Wong PTP, Wong LCJ (2006). Coping strategies and culturally influenced beliefs about the world. International and cultural psychology.

[24] Dib S, Rougeaux E, Vázquez-Vázquez A, Wells JC, Fewtrell M (2020). Maternal mental health and coping during the COVID-19 lockdown in the UK: data from the COVID-19 new mum study. Int J Gynecol Obstet.

[25] Saccone G, Florio A, Aiello F, Venturella R, De Angelis MC, Locci M (2020). Psychological impact of coronavirus disease 2019 in pregnant women. Am J Obstet Gynecol.

[26] Berthelot N, Lemieux R, Garon-Bissonnette J, Drouin-Maziade C, Martel É, Maziade M (2020). Uptrend in distress and psychiatric symptomatology in pregnant women during the coronavirus disease 2019 pandemic. Acta Obstet Gynecol Scand.

[27] Patabendige M, Gamage MM, Weerasinghe M, Jayawardane A (2020). Psychological impact of the COVID-19 pandemic among pregnant women in Sri Lanka. Int J Gynecol Obstet.

[28] Hamzehgardeshi Z, Omidvar S, Amoli AA, Firouzbakht M (2021). Pregnancy-related anxiety and its associated factors during COVID-19 pandemic in Iranian pregnant women: a web-based cross-sectional study. BMC Pregnancy Childbirth.

[29] Bjelica A, Cetkovic N, Trninic-Pjevic A, Mladenovic-Segedi L (2018). The phenomenon of pregnancy — a psychological view. Ginekol Pol.

[30] Cohen J (1988). Statistical power analysis for the behavioral sciences.

[31] Rasouli P, Shobeiri F, Cheraghi F, Rasouli R, Ghanbari V (2016). Study of the relationship of anxiety and depression in third trimester pregnancy on growth index of neonates and preterm delivery. Iran J Pediatr.

[32] Ahmadi kani Golzar A, GoliZadeh Z (2015). Validation of Edinburgh postpartum depression scale (EPDS) for screening postpartum depression in Iran. Iranian journal of psychiatric. Nursing.

[33] Alipour A, Ghadami A, Alipour Z, Abdollahzadeh H (2020). Preliminary validation of the Corona disease anxiety scale (CDAS) in the Iranian sample. Q J Health Psychol.

[34] Carver CS (1997). You want to measure coping but your protocol’too long: consider the brief cope. Int J behav Med.

[35] Cooper C, Katona C, Orrell M, Livingston G (2008). Coping strategies, anxiety and depression in caregivers of people with Alzheimer's disease. Int J Geriatr Psychiatry.

[36] Ashktorab T, Baghcheghi N, Seyedfatemi N, Baghestani A (2017). Psychometric parameters of the Persian version of the BriefCOPE among wives of patients under hemodialysis. Med J Islam Repub Iran.

[37] Rahimi R, Dolatabadi Z, Moeindarbary S, Behzadfar S, Fakhr Ghasemi N, Tafrishi R (2020). A systematic review of the prevalence of mental health disorders in pregnant women during the COVID-19 pandemic. Int J Pediatr.

[38] Wang C, Horby PW, Hayden FG, Gao GF (2020). A novel coronavirus outbreak of global health concern. Lancet (London, England).

[39] Tomfohr-Madsen LM, Racine N, Giesbrecht GF, Lebel C, Madigan S (2021). Depression and anxiety in pregnancy during COVID-19: a rapid review and meta-analysis. Psychiatry Res.

[40] Huizink AC, de Medina PGR, Mulder EJ, Visser GH, Buitelaar JK (2002). Coping in normal pregnancy. Ann Behav Med.

[41] Li Q (2020). Psychosocial and coping responses toward 2019 coronavirus diseases (COVID-19): a cross-sectional study within the Chinese general population. QJM.

[42] Guo J, Feng XL, Wang XH, MH v IJ (2020). Coping with COVID-19: exposure to COVID-19 and negative impact on livelihood predict elevated mental health problems in Chinese adults. Int J Environ Res Public Health.

[43] AlHadi AN, Alarabi MA, AlMansoor KM (2021). Mental health and its association with coping strategies and intolerance of uncertainty during the COVID-19 pandemic among the general population in Saudi Arabia: cross-sectional study. BMC Psychiatry.

[44] Fan S, Guan J, Cao L, Wang M, Zhao H, Chen L, Yan L. Psychological effects caused by COVID-19 pandemic on pregnant women: a systematic review with meta-analysis. Asian J Psychiatr. 2021;56:102533. 10.1016/j.ajp.2020.102533 PMC7833174 33418283

[45] Hessami K, Romanelli C, Chiurazzi M, Cozzolino M. COVID-19 pandemic and maternal mental health: a systematic review and meta-analysis. J Matern Fetal Neonatal Med. 2020:1–8. 10.1080/14767058.2020.1843155 33135523

[46] Lau Y, Wang Y, Kwong D (2015). Are different coping styles mitigating perceived stress associated with depressive symptoms among pregnant women?. Perspect Psychiatr Care.

[47] Jungmann SM, Witthöft M (2020). Health anxiety, cyberchondria, and coping in the current COVID-19 pandemic: which factors are related to coronavirus anxiety?. J Anxiety Disord.

[48] Faisal-Cury A (2012). Coping style and depressive symptomatology during pregnancy in a private setting sample. Spanish J Psychol.

[49] Blechman EA, Lowell ES, Garrett J (1999). Prosocial coping and substance use during pregnancy. Addict Behav.

[50] Hamilton JG, Lobel M (2008). Types, patterns, and predictors of coping with stress during pregnancy: examination of the revised prenatal coping inventory in a diverse sample. J Psychosom Obstet Gynecol.

[51] Rodriguez CM (2009). Coping style as a mediator between pregnancy desire and child abuse potential: A brief report. J Reprod Infant Psychol.

[52] Latendresse G (2009). The interaction between chronic stress and pregnancy: preterm birth from a biobehavioral perspective. J Midwifery Womens Health.

[53] Taylor SE, Stanton AL (2007). Coping resources, coping processes, and mental health. Annu Rev Clin Psychol.

[54] Latendresse G, Ruiz RJ (2010). Maternal coping style and perceived adequacy of income predict CRH levels at 14-20 weeks of gestation. Biol Rese Nurs.

[55] Wheeler JM, Misra DP, Giurgescu C. Stress and coping among pregnant black women during the COVID-19 pandemic. Public Health Nurs. 2021. 10.1111/phn.12909 PMC8250999 33844868

